# Altered hippocampal volume and functional connectivity in patients with Cushing's disease

**DOI:** 10.1002/brb3.2507

**Published:** 2022-05-04

**Authors:** Chuqi Li, Yanyang Zhang, Wenxin Wang, Tao Zhou, Xinguang Yu, Hong Tao

**Affiliations:** ^1^ Department of Neurosurgery The First Affiliated Hospital of Nanchang University Nanchang China; ^2^ Department of Neurosurgery Chinese PLA General Hospital Beijing China; ^3^ Department of Radiology The First Medical Center of Chinese PLA General Hospital Beijing China

**Keywords:** Cushing's disease, glucocorticoids, hippocampus, MRI

## Abstract

**Introduction:**

Stress‐related brain disorders can be associated with glucocorticoid disturbance and hippocampal alteration. However, it remains largely unknown how cortisol affects the structure and function of hippocampus. Cushing's disease (CD) provides a unique “hyperexpression model” to explore the effects of excessive cortisol on hippocampus as well as the relation between these effects and neuropsychological deficits.

**Methods:**

We acquired high‐resolution T1‐weighted and resting‐state functional magnetic resonance imaging in 47 CD patients and 53 healthy controls. We obtained the volume and functional connectivity of the hippocampal rostral and caudal subregions in both groups. Relationships between hippocampal alterations, neuroendocrine, and neuropsychological assessments were identified.

**Results:**

Relative to control subjects, the CD patients had smaller volumes of all four hippocampal subregions. Furthermore, whole brain resting‐state functional connectivity analyses with these four different hippocampal regions as seeds revealed altered hippocampal functional connectivity with high‐order networks, involving the DMN, frontoparietal, and limbic networks in CD patients. The intrinsic hippocampal functional connectivity was associated with the quality of life of the CD patients.

**Conclusions:**

Our findings elucidate the cumulative effect of excess cortisol on the morphology and function of hippocampus and reinforce the need for effective interventions in stress‐related brain disease to halt potential hippocampal damage.

## Graphical abstract

1. CD patients had significant atrophy of all four hippocampal subregions.

2. Altered hippocampal functional connectivity with high‐order networks was found in CD patients.

3. Effective interventions are needed to halt potential hippocampal damage in stress‐related brain disease.

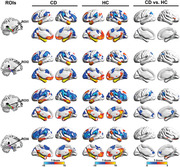

## 1 INTRODUCTION

Converging evidence has pointed to a strong linkage between the cortisol and human brain and stress‐related neuropsychiatry disorders, such as major depression disorder and posttraumatic stress disorder (de Kloet et al., 2005). However, it remains to be established how this stress hormone influences specific brain structures and functions, particularly in humans, which is of particular importance for both treatment of stress‐related disorders and research on cortisol effects in the brain.

Cushing's disease (CD) is caused by an adrenocorticotropic hormone pituitary adenoma and characterized by chronic hypercortisolism. This condition is therefore a unique and natural “hyperexpression model” to investigate the chronic effects of cortisol on brain physiology and cognition (Zhang et al., 2021). By applying multimodal neuroimaging techniques to CD patients, previous studies have observed that chronic hypercortisolism could cause a number of abnormalities in various brain phenotypes. Among these neural changes of CD patients, hippocampal anomalies are the most replicated findings. Studies on CD patients report hippocampal changes that converge with morphologic alterations such as reduction in volume (Burkhardt et al., 2015; Toffanin et al., 2011). Moreover, abnormal cerebral blood flow and glucose metabolism in hippocampus have also been found in CD patients. Both structural and functional alterations in the hippocampus might contribute to the psychotic symptoms in CD patients (Frimodt‐Møller et al., 2019). However, it is well established that psychosis is better described as a brain connectional diaschisis rather than isolated regional dysfunctions (Matthews & Hampshire, 2016). These current hippocampus‐related findings were mainly obtained by voxel‐based or regional analyses of brain volume or metabolism properties, and researchers have not determined whether the organizational patterns of hippocampal functional connectivity are disrupted in CD patients.

The hippocampus is easily targeted by long‐term hypercortisolism because this area is a part of the stress response system and is abundant in mineralocorticoid receptors and glucocorticoid receptors (McEwen et al., 2016). Also recently, studies on macaques and humans have observed that hippocampus is an anatomically and functionally heterogeneous region along the rostral/caudal‐dorsal/ventral axis (Schultz & Engelhardt, 2014). Specifically, the rostral hippocampus has connections with prefrontal regions and relates to stress, emotion, and affect. In contrast, the caudal hippocampus mainly connects to sensory cortical areas and performs primarily cognitive functions (Fanselow & Dong, 2010). Therefore, the hippocampus should be studied in a set of separate structures with rostral and caudal hippocampus. Whether the hippocampal subregions exhibit differentially altered connectivity patterns responding to chronic hypercortisolism remains largely unknown.

The present study further extends this work by examining the relationship between hippocampal subregions and resting‐state functional connectivity in large‐scale brain networks, as measured by resting‐state fMRI (rs‐fMRI) (Park & Friston, 2013). We focus on default mode network (DMN), frontoparietal, and limbic networks, given their involvement in stress related psychiatric illnesses. The first is the DMN, which supports self‐related cognitive functions. Complementing the DMN is the frontoparietal network, which supports the cognitive regulation of behavior and emotion. Finally, the limbic networks play a key role in emotion regulation.

In this study, first, to explore the structural changes of hippocampal subregions in CD patients, we performed a volumetric MRI analysis of the four subregions (left rostral hippocampus, left caudal hippocampus, right rostral hippocampus, and right caudal hippocampus). Given the known direct neurotoxic effects of cortisol on hippocampus, we predicted that chronic hypercortisolism caused smaller hippocampal volumes in CD patients. Second, we used these four subregions as seed regions separately and mapped whole‐brain functional connectivity patterns associated with each subregion to examine alterations in hippocampal functional connectivity in CD patients. Considering the psychiatric symptoms in CD patients, it is reasonable to expect the presence of altered hippocampal functional connectivity with high‐order networks.

## 2 MATERIAL AND METHODS

### 2.1 Participants

A total of 47 participants with a diagnosis of CD and 53 healthy control (HC) subjects were included in this study. The CD patients underwent transsphenoidal surgery at the Department of Neurosurgery, The First Medical Center of Chinese People's Liberation Army (PLA) General Hospital between May 2017 and November 2019. According to the clinical practice guideline (Nieman et al., 2015), CD was diagnosed by experienced endocrinologists and confirmed by postsurgical pathology. The detailed preoperative assessments of diagnostic criteria have been reported in our previous study. HCs were recruited from the local community and were controlled for any history of psychopathology abnormalities. All participants were right‐handed and had normal vision and auditory sensation. The study was approved by the local ethics committee of the Chinese PLA General Hospital and written informed consent was obtained from each participant. The data of these 47 CD and 53 HC subjects have been partially used in our previous studies (Wang et al., 2019; Zhang et al., 2021).

### 2.2 Neuroendocrine and neuropsychological assessment

All participants underwent biochemical evaluation to assess their cortisol level. We quantified the levels of 24‐h urinary free cortisol (24hUFC, nmol/24h); serum cortisol (nmol/L) at 0:00, 8:00, and 16:00. Cortisol was detected with an ADVIA Centaur Analyzer (Siemens Healthcare Diagnostics, Tarrytown, NY, USA). Cortisol levels at 8:00 as well as 24hUFC were also measured in 51 HC subjects.

All participants underwent a comprehensive neuropsychological assessment with an expert psychiatrist, including Self‐Rating Depression Scale (SDS), Self‐Rating Anxiety Scale (SAS), Mini‐mental State Examination (MMSE), and Montreal Cognitive Assessment (MoCA). Moreover, health‐related quality of life and neuropsychiatric symptoms of CD patients were evaluated with the Cushing's Quality‐of‐Life (CushingQoL) questionnaire (Nelson et al., 2013) and Chinese version of the neuropsychiatric inventory (CNPI) (Leung et al., 2001), respectively.

### 2.3 Image acquisition

Structural and functional MRI data were acquired on a 3.0‐Tesla MR system (Discovery MR750, General Electric) with an 8‐channel head coil. High‐resolution structural 3D T1‐weighted images were conducted using a sagittal Fast Spoiled Gradient‐Echo (FSPGR) sequence with the following parameters: repetition time = 6.7 ms, echo time = 2.9 ms, flip angle = 7°, field of view = 250 × 250 mm^2^, number of slices = 192, voxel size = 1 × 1 × 1 mm^3^ with no gap. The functional images were acquired using an echo‐planar imaging (EPI) sequence with repetition time = 2000 ms, echo time = 30 ms, flip angle = 90°, thickness/gap = 3.5 mm/0.5 mm, slices = 36, field of view = 224 × 224 mm^2^, voxel size = 3.5 × 3.5 × 3.5 mm^3^, number of total volumes = 240. Soft earplugs were used to attenuate scanner noise and head motion was restrained with foam padding. During functional scanning, all participants were requested to keep their eyes closed and stay awake.

### 2.4 rs‐fMRI data preprocessing

Preprocessing of the rs‐fMRI images was conducted using SPM12 and Data Processing Assistant for Resting‐State fMRI (DPABI, http://www.restfmri.net/forum/DPARSF). The first 10 volume of the functional images were removed to avoid initial steady‐state problems. Then functional images were spatially realigned to the first image for motion correction, and reslicing for acquisition temporal delay. The head motion of all participants in this study had no more than 2‐mm translation or 2° rotation in any direction. Next, functional images were coregistered to each participant's segmented gray matter T1 image, and then spatially normalized to the MNI space, resampled to 3‐mm isotropic voxels. Subsequently, the global signal, white matter signal, cerebrospinal fluid signal and 24‐motion vectors were regressed from the data. Finally, linear detrending and bandpass filter (0.01−0.08 Hz) were carried out to reduce the effects of low‐frequency drift and high‐frequency physiological noise.

### 2.5 Hippocampal functional connectivity

The hippocampus has been functionally parcellated into four subregions (left rostral hippocampus, left caudal hippocampus, right rostral hippocampus, and right caudal hippocampus) based on Human Brainnetome Atlas (Fan et al., 2016). On each hippocampal subregion, we performed seed‐based functional connectivity analysis. Briefly, hippocampal functional connectivity maps were obtained by computing the Pearson correlation coefficient for each voxel's time course with the average time course inside the region of interest. Notably, the computation was constrained within a gray‐matter mask which was generated by thresholding (a threshold of 0.2) a prior gray‐matter probability map in SPM12. The resulting correlation coefficients were further converted to *z* scores using Fisher's *r*‐to‐*z* transform to improve normality. For each subject, we obtained 4 *z*‐score maps indicative of the intrinsic functional connectivity patterns of the four hippocampal subregions. To exclude the possible confounding effect of hippocampal volume in CD patients, we performed a voxel‐based morphometry analysis on structural MRI images and took the volume of hippocampal subregions as a covariate in the functional connectivity statistical analyses.

### 2.6 Statistical analysis

All demographic and clinical variables including neuroendocrine and neuropsychological scores were compared by two‐sample *t*‐tests. Sex composition of the two groups was compared using a Pearson's chi‐square test (two‐tailed). To explore differences in hippocampal functional connectivity between CD patients and HCs, general linear models were performed in a voxel‐wise fashion. To exclude the possible confounding effects of age, gender, education level, and volume of hippocampal subregions, we used these measures as covariates in the general linear models. Multiple comparison correction was performed using a FDR of 0.05 within the grey matter mask.

In CD patients group, a linear regression analysis was further performed to explore the relationship between functional connectivity of the clusters showing significant group differences and neuropsychological scores as well as the endocrinological indicators (cortisol and 24hUFC). Multiple comparisons were also corrected using the FDR method with a corrected threshold of *q* < 0.05.

## 3 RESULTS

### 3.1 Demographic, endocrinological, and neuropsychological results

Table 1 shows the demographic characteristics of the CD patients and the HCs. There were no significant differences in terms of age, sex distribution, and years of education between groups. Compared with HCs, CD patients had significantly lower MoCA scores and higher SDS and SAS scores (Table 1). As expected, the CD patients had significantly higher levels of serum cortisol and 24hUFC (*p* < .001). Moreover, we calculated the volumes of the four hippocampal subregions and found smaller volumes of all four hippocampal subregions in the CD patients.

**TABLE 1:** Participant characteristics

	CDs (*n* = 47)	HCs (*n* = 53)	*p* Value
Age (years)	37.38 ± 10.61 (20–59)	34.79 ± 10.72 (21–63)	.113
Gender (male/female)	4/43	4/49	.859
Education (years)	11.00 ± 4.11	11.74 ± 3.10	.311
Illness duration (months)	41.62 ± 53.71	—	—
Neuropsychological tests			
MoCA	22.47 ± 3.98 (*n* = 45)	27.72 ± 2.00	<.001
SDS	40.18 ± 9.96 (*n* = 45)	27.13 ± 4.42	<.001
SAS	38.27 ± 7.90 (*n* = 45)	26.98 ± 4.47	<.001
CNPI	11.93 ± 9.68 (*n* = 45)	—	—
Cushing QOL	37.76 ± 8.29 (*n* = 45)	—	—
Endocrinological tests			
Serum cortisol (nmol/L)			
0:00 am	633.81 ± 237.59 (*n* = 46)	—	—
8:00 am	735.34 ± 279.44 (*n* = 47)	358.51 ± 107.43 (*n* = 51)	<.001
16:00 pm	671.05 ± 273.56 (*n* = 47)	—	—
24hUFC (nmol/24 h)	2381.59 ± 1653.16 (*n* = 41)	252.03 ± 119.47 (*n* = 47)	<.001
Volume of hippocampal subregions (mm^3^)			
Left rostral hippocampus	343.75 ± 39.15 (257.18–423.27)	365.69 ± 27.19 (313.21–442.06)	.001
Left caudal hippocampus	272.69 ± 32.74 (206.63–339.04)	296.39 ± 23.13 (249.62–347.61)	<.001
Right rostral hippocampus	305.10 ± 33.71 (229.67–396.89)	336.76 ± 25.98 (274.95–415.16)	<.001
Right caudal hippocampus	320.42 ± 32.60 (238.16–396.58)	347.87 ± 27.16 (294.00–415.80)	<.001

Abbreviations: 24hUFC, 24‐h urinary free cortisol.; CDs, Cushing's disease patients; CNPI, Chinese version of neuropsychiatric inventory; Cushing QOL, Cushing Quality of Life Scale; HCs, healthy controls; MoCA, Montreal Cognitive Assessment; SAS, Self‐Rating Anxiety Scale; SDS, Self‐Rating Depression Scale.

*Note*: All values are expressed as mean ± SD. Group differences in sex between CDs and HCs were examined using chi‐square test. Group differences in the other demographic and clinical characteristics between CDs and HCs were examined using two‐sample *t*‐tests (two‐tailed).

### 3.2 Spatial distribution of hippocampal functional connectivity

**FIGURE 1:**
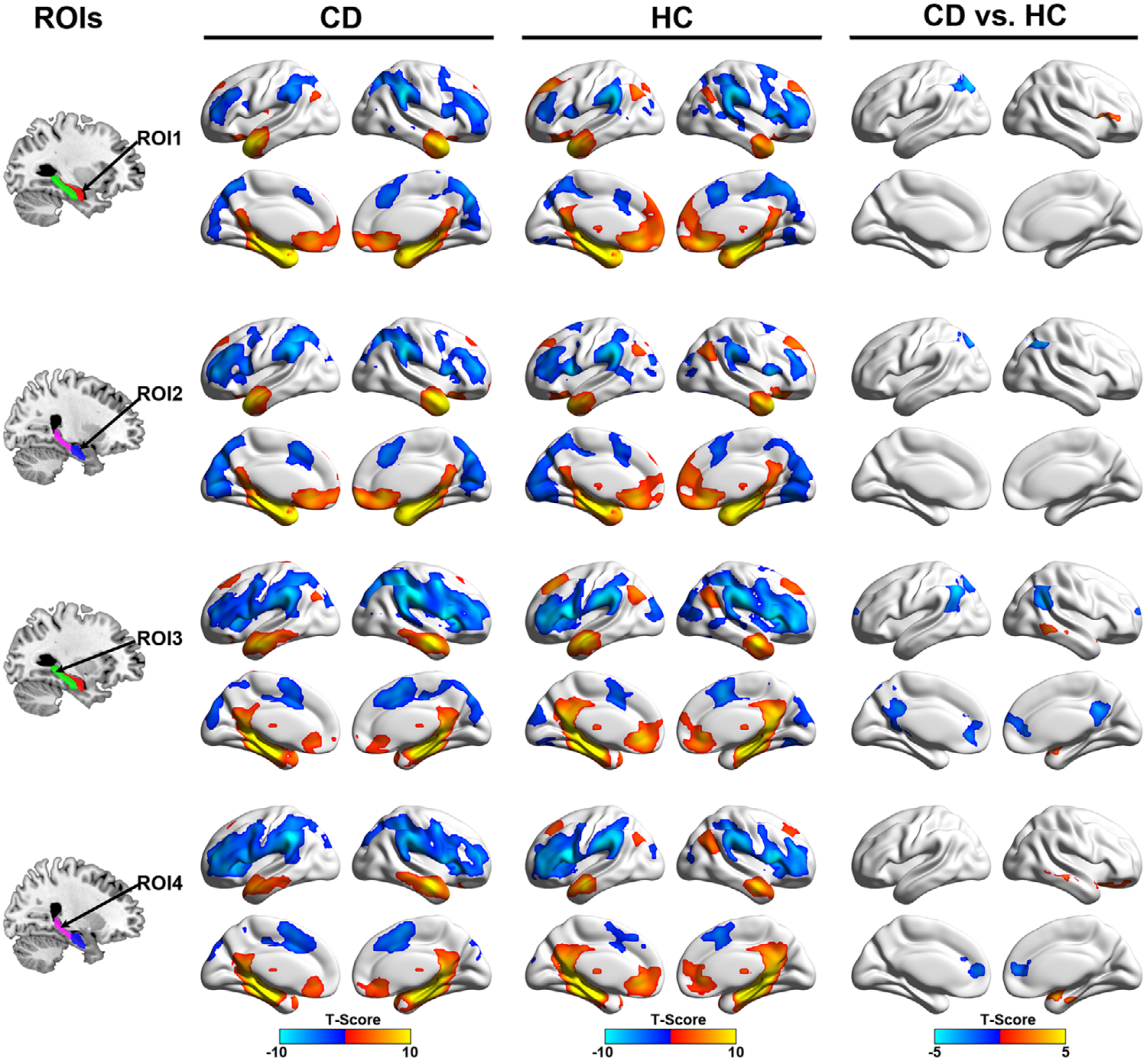
Between‐group differences in functional connectivity of the hippocampal subregions. The first column shows the hippocampal functional connectivity subregions. The second and third columns show the hippocampal functional connectivity maps within CD and HC groups, respectively. Further between‐group comparisons showed that CD patients had significantly altered hippocampal functional connectivities relative to HCs, with a corrected statistical threshold of *p *< .05. ROI1, left rostral hippocampus; ROI2, left caudal hippocampus; ROI3, right rostral hippocampus; ROI4, right caudal hippocampus; ROI, region of interest; CD, Cushing's disease; HC, healthy control

The hippocampal functional connectivity maps of both CD patients and HCs are presented in Figure 1. Visually, the spatial distributions of hippocampal functional connectivity were highly similar between groups, in spite of some differences in strength. We observed that the brain regions significantly positively connecting to hippocampus were primarily distributed in several limbic network regions (the orbital frontal cortex, bilateral medial temporal regions, and temporal pole) and DMN regions (bilateral medial frontal cortex, posterior cingulate gyrus/precuneus, and anterior cingulate cortex). Brain regions with negative connectivity to hippocampus were chiefly distributed in the frontoparietal network regions (dorsolateral prefrontal cortex, supramarginal gyrus, and angular gyrus).

### 3.3 Altered hippocampal functional connectivity in CD patients

The significant differences in functional connectivity with each hippocampal subregion between the CD patients and HCs groups are illustrated in third column of Figure 1. Both the right and left rostral hippocampus exhibited significantly decreased functional connectivity with the superior parietal lobe (SPL), a component of the frontoparietal network. Moreover, right rostral hippocampus exhibited additional increased functional connectivity with right inferior frontal gyrus, a component of DMN. For the left caudal hippocampus, significantly altered functional connectivity was found to the DMN regions, including (bilateral medial frontal cortex, angular gyrus, anterior, and posterior cingulate cortex). We also observed decreased functional connectivity between the right caudal hippocampus and anterior cingulate cortex. Additionally, the right caudal hippocampus exhibited increased functional connectivity with some limbic regions including the right orbital frontal cortex and temporal pole (Table 2).

**TABLE 2:** Brain regions showing changed RSFC between CDs and HCs groups

				Peak MNI coordinate			
	Brain regions	BA	Cluster size (voxels)	*x*	*y*	*z*	Peak *T*
ROI‐based RSFC							
ROI1	R IFG	48	219	57	21	—3	4.598
	L angular	39	423	−27	−72	51	−5.530
RIO2	R thalamus	−	114	9	−6	3	−5.905
	L angular	39	195	−27	−72	54	−4.830
	R angular	39	384	36	−66	48	−5.607
ROI3	R MTG	20	633	39	6	−21	4.410
	L angular	39	195	−27	−72	54	−4.830
	R angular	39	384	36	−66	48	−5.607
	MFG/ACC	10/32	572	−3	42	−3	−4.033
	PCC/PreCUN	26/23	709	12	−45	27	−4.502
ROI4	MFG/ACC	32	465	3	48	6	−4.670
	R MTG/OFC	48/21	747	30	3	−21	4.208

*Note*: Statistical threshold was set at *p* < .05, corrected.

Abbreviations: CDs, Cushing's disease patients; HCs, healthy controls; ROI, regions of interest; BA, Brodmann areas; MNI, Montreal Neurological Institute; RSFC, resting‐state functional connectivity; SFG, superior frontal gyrus; MFG, middle frontal gyrus; dMFG, dorsal medial frontal gyrus; IPL, inferior parietal lobule; AG, angular gyrus; ROL, rolandic operculum; Ins, insular; PrCG, precentral gyrus; L, left; R, right; ROI1, left rostral hippocampus; ROI2, left caudal hippocampus; ROI3, right rostral hippocampus; ROI4, right caudal hippocampus.

### 3.4 Brain–behavior relationships in the CD patients

**FIGURE 2:**
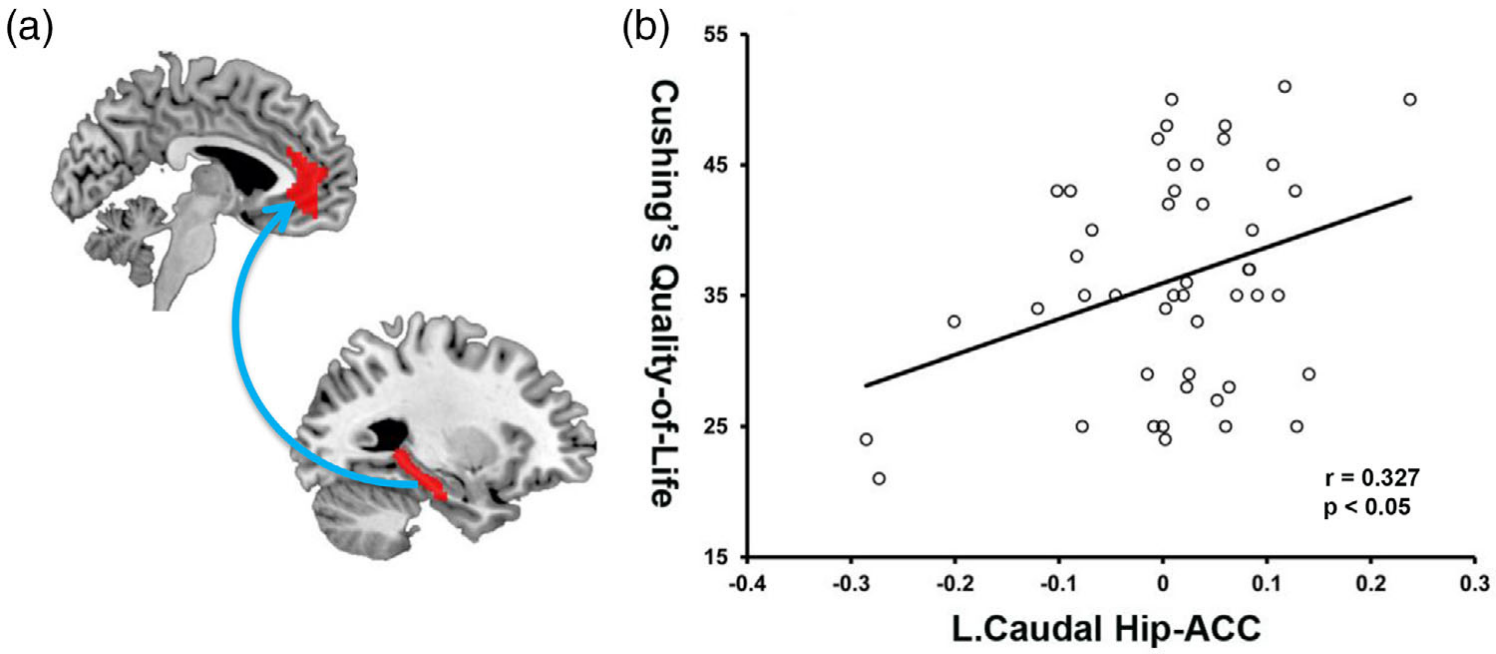
Significant correlations between left hippocampal functional connectivity and the quality of life in CD patients. CD, Cushing's disease; Hip, hippocampus; ACC, anterior cingulate cortex

In the correlation analyses of CD patients, the mean values of the functional connectivity between the left caudal hippocampus and anterior cingulate cortex correlated positively with the Cushing's QoL scores (*r* = .327, *p* < .05) (Figure 2). No other correlations were found for volumes and functional connectivity of the four hippocampal subregions with neuroendocrine and neuropsychological assessment in the CD patients.

## 4 DISCUSSION

Using a cohort of CD patients and HCs, the present study performed a comprehensive investigation to reveal how the chronic hypercortisolism affects the morphology and connectivity of hippocampal subregions and their relationships with neuroendocrine and neuropsychological assessment. Compared with the HCs, the CD patients had smaller volumes of all four hippocampal subregions. Furthermore, CD patients exhibited differential patterns of altered hippocampal functional connectivity with high‐order networks, involving the DMN, frontoparietal, and limbic networks. The intrinsic hippocampal functional connectivity was associated with the quality of life of the CD patients. Together, these findings elucidate the cumulative effect of cortisol on the morphology and function of hippocampus and provide important information to further understand the role of hippocampus in stress‐related brain disease.

Cortisol, the end product of the hypothalamic–pituitary–adrenal axis, plays a critical role in the body's response to stress and maintenance of homeostasis (Sapolsky et al., 2000); however, chronic hypercortisolism is known to impair neurons in the hippocampus. CD patients naturally demonstrate chronic excessive amounts of cortisol; therefore these patients serve as a natural “hyperexpression model” to investigate the chronic effects of cortisol on human hippocampus. Importantly, we showed the CD patients are associated with smaller hippocampal volumes in all four subregions. In line with our study, previous structural imaging studies have shown hippocampal volume decreases in CD patients (Frimodt‐Møller et al., 2019; Toffanin et al., 2011). Furthermore, Brown et al. found that healthy volunteers were associated with a significant reduction in hippocampal volume following only 3‐day stress doses of corticosteroid administration, strongly suggesting the effects of cortisol on hippocampal size. It is important to note that chronic hypercortisolism can affect the hippocampus in at least two ways: by direct neurotoxic effects on the hippocampus (Lupien et al., 2018; Uno et al., 1994) and by reduction in hippocampal neurogenesis (Saaltink & Vreugdenhil, 2014). Moreover, cortisol stimulates the release of excitatory amino acids glutamate on hippocampal cells (de Kloet et al., 2005). On the other hand, chronic elevations of cortisol also reduce neurotrophic factors that includes nerve growth factor and brain‐derived neurotrophic factor (McEwen et al., 2015).

The different patterns of functional connectivity in rostral hippocampus versus caudal hippocampus might be associated to the specific cytoarchitecture along the rostral/caudal hippocampus. Accumulated evidence from both animal and human studies suggests that different parts of the hippocampus display distinctive gene expression and anatomical projections patterns (Fanselow & Dong, 2010). In detail, gene expression in the rostral hippocampus correlates with regions involved in emotion and stress (amygdala and hypothalamus). Moreover, the rostral hippocampus has connections with prefrontal regions, exerts strong regulatory control of the hypothalamic–pituitary–adrenal axis with a negative feedback (Toffanin et al., 2011). Accordingly, as demonstrated in this study, chronic hypercortisolism predominantly disrupted the functional connectivity in rostral hippocampus.

Another major finding in this study was altered hippocampal functional connectivity with DMN, frontoparietal, and limbic networks in CD individuals relative to that in HCs. Emerging evidence proposes that interactions within and between these large‐scale brain networks play important roles on brain functions and may be affected in multiple psychiatric disorders (Menon, 2011; Sha et al., 2019). Among these brain networks, the DMN is anchored in the medial prefrontal cortex and posterior cingulate cortex and is implicated in internally directed attention and self‐referential processing (Raichle, 2015), while the frontoparietal and limbic networks support the cognitive regulation of emotion, attention, and behavior (Buhle et al., 2014; Kohn et al., 2014). The engagement of these high‐level functional networks may suggest the linkage of abnormal stress hormone cortisol to cognitive deficits in CD patients. In line with our study, previous studies have shown stress‐induced cortisol increase was associated with altered connectivity within the major brain networks (Zhang et al., 2019, 2020, 2020). Meanwhile, structural and functional alterations in these brain systems are also found in CD patients. For example, many functional imaging studies have consistently demonstrated altered brain activities and functional connectivity involving in DMN, frontoparietal, and limbic networks (Jiang et al., 2017; Wang et al., 2019; Zhang et al., 2021), even in the patients with long‐term remission of CD (van der Werff et al., 2015). Importantly, previous studies have shown that the CD patients had widespread reductions of white matter integrity, which provide further evidence for the structural substrate for the persistence of these functional deficits (Pires et al., 2015; van der Werff et al., 2014). Here, we propose that by altering hippocampal processes via the abundant glucocorticoid and mineralocorticoid receptors, exposure to hypercortisolism disrupts the interactions with DMN, frontoparietal, and limbic networks in CD patients, thus engender vulnerability for emotional and cognitive problems. In line with this view is evidence that altered hippocampal functional connectivity is associated with the quality of life in CD patients. Because impaired quality of life is a persistent complaint from CD patients (Webb et al., 2018), it is important to accurately assess which aspects of QoL are affected in order to better understand the severity of hypercortisolism on patients and the potential efficacy of treatment. CushingQoL questionnaire has proven to be a valuable resource for assessing health‐related QoL in CD patients, based on the combination of psychosocial issues and physical problems (Nelson et al., 2013). A better understanding of the neuroplasticity and continuing quality of life change may in turn facilitate advances in management and intervention.

Several issues need to be addressed further. First, although the sample size of this study was relatively large, the findings still need to be further replicated in an independent sample. Second, the cross‐sectional, observational nature of our study design precludes any causal conclusions. Therefore, studies tracking dynamic changes in hippocampal functional connectivity following the remission of hypercortisolism are needed. We are currently following up participants as part of a longitudinal study. Finally, a combined analysis of multimodal imaging including structural and metabolic data would provide integrated information on the effect of cortisol excess on human brain.

In short, we demonstrate that CD patients present atypical morphology and functional connectivity of hippocampus. Here we observed the chronic hypercortisolism caused smaller volumes of all hippocampal subregions. This volume change was in line with the preclinical research that excess cortisol cause dendritic shrinkage and loss of spines in the hippocampus. Functionally, CD patients demonstrated altered hippocampal connectivity whose nodes include key components of the DMN, frontoparietal, and limbic networks. These multimodal results reinforce the need for effective therapeutic interventions in stress‐related brain disease to halt possible hippocampal damage.

## CONFLICT OF INTEREST

The authors report no biomedical financial interests or potential conflicts of interest.

### PEER REVIEW

The peer review history for this article is available at https://publons.com/publon/10.1002/brb3.2507

## Data Availability

Data that support the findings of this study and custom code used to analyze data are available from the corresponding author upon reasonable request.
